# Exosomal Cripto-1 Serves as a Potential Biomarker for Perihilar Cholangiocarcinoma

**DOI:** 10.3389/fonc.2021.730615

**Published:** 2021-08-09

**Authors:** Chunxiao Hu, Yanli Zhang, Mengjiao Zhang, Tingting Li, Xin Zheng, Qining Guo, Xin Zhang

**Affiliations:** ^1^Department of General Surgery, Qilu Hospital of Shandong University, Jinan, China; ^2^Department of Clinical Laboratory, Shandong Provincial Third Hospital, Jinan, China; ^3^Department of Clinical Laboratory, Qilu Hospital of Shandong University, Jinan, China

**Keywords:** exosomes, biomarker, diagnosis, prognosis, perihilar cholangiocarcinoma, Cripto-1

## Abstract

Perihilar cholangiocarcinoma (PHCCA) has a poor prognosis, mainly due to diagnosis at an advanced stage. Cripto-1 functions as an oncogene and is highly expressed in several human cancers, however, its clinical application in PHCCA is poorly understood. Herein, we identified that Cripto-1 was released by PHCCA cells *via* exosomes *in vitro* and *in vivo*. Furthermore, an ELISA method was developed to detect exosomal Cripto-1 in the serum of 115 PHCCA patients, 47 cholangitis patients and 65 healthy controls, and it was found that exosomal Cripto-1 was increased in PHCCA patients and associated with metastasis. Compared with traditional serum tumor markers, CA19-9 and CEA, exosomal Cripto-1 demonstrated a larger area under ROC curve for PHCCA diagnosis. The cutoff value of exosomal Cripto-1 was 0.82, achieving a sensitivity of 79.1% and a specificity of 87.5%. As expected, exosomal Cripto-1 levels in immunohistochemically Cripto-1-high cases were significantly elevated compared to in Cripto-1-low cases. When measured 1-week postoperatively, Cripto-1 levels decreased on average from 1.25(0.96-3.26) to 0.85(0.62-1.82). Immunohistochemistry analysis showed Cripto-1 expression was negatively correlated with E-cadherin and was an independent prognostic biomarker for poor survival in PHCCA patients. In conclusion, exosomal Cripto-1 in sera can reflect its expression in the tissue of PHCAA patients and has the potential be a non-invasive biomarker for diagnosis and prognosis of PHCCA.

## Introduction

Perihilar cholangiocarcinoma (PHCCA), also known as a Klatskin tumor, is a subtype of CCA, arising at or near the confluence of the right and left hepatic duct. It accounts for more than 50% of total CCA cases and is characterized by high surgical difficulty and poor prognosis (1–3). Even for those patients who undergo curative intent resection, the 5-year overall survival rate is only about 30% (4). Moreover, due to a lack of clear symptoms, most PHCCA patients are diagnosed at an advanced stage at which surgical treatment is not a viable option (5). If PHCCA could be diagnosed before jaundice or intrahepatic biliary dilatation on imaging, patients might have better outcomes (6). However, until now there are no reliable biomarkers for early diagnosis of PHCCA.

Cripto-1, also known as teratocarcinoma-derived growth factor1 (TDGF-1), is a member of the epidermal growth factor/Cripto-1-FRL-1-Cryptic family (7). It is isolated and cloned from the human teratocarcinoma cDNA library of NTERA-2, and is indispensable for early embryonic development and maintenance of the pluripotency of embryonic stem cells (8). Recently, Cripto-1 has been reported to be an oncogene that plays an important role in the initiation and progression of several types of human cancers (9). Some studies have shown that Cripto-1 is involved in the epithelial-mesenchymal transition (EMT), whereby it enhances the invasion and metastasis of tumor cells (10, 11). Cripto-1 can also trigger mitogen activated protein kinase and Akt signaling pathways, to promote cell survival and migration *via* specific binding to Glypican-1 (12). In addition, overexpression of Cripto-1 enhances the proliferation of human endothelial cells. The subsequent increase in microvessel formation levels was detected by a directed angiogenesis assay *in vivo*, which confirmed the role of Cripto-1 in regulating tumor angiogenesis (13). Simultaneously, inhibition of Cripto-1 expression, by anti-Cripto-1 antibody (14) or microRNA-15b (15), exhibits a potential to suppress growth of cancer cells. Thus, Cripto-1 may represent a novel molecular target for diagnosis and prognosis of cancers.

Exosomes are cell-secreted bilayered membrane vesicles with a diameter of 30-120 nm (16). They originate from multivesicular bodies(MVB)/late endosomes inside the cell and are released into the external space upon fusion of MVBs with the cell membrane (17). Until now, it has been well-documented that almost all tumor cells secrete exosomes, and the presence of exosomes has been confirmed in a wide variety of body fluids, such as plasma/serum, urine, saliva, and bile (18). Moreover, it has been reported that cancer cell-secreted exosomes can be transferred to recipient cells and promote tumor progression by enhancing immunosuppression, angiogenesis and metastatic dissemination (19). More importantly, these extracellular vesicles contain specific components, such as lipids, proteins, and nucleic acids, mirroring their cellular origin (20). Thus, it is feasible that detection of enriched and specific molecules in cancer exosomes could serve as a liquid biopsy to aid in the diagnosis of malignancies. For instance, CKAP4 is detected in exosomes harvested from the serum of patients with pancreatic cancer and reflects its expression in tumor lesions, which may represent a biomarker for pancreatic cancer (21). However, whether Cripto-1 can be released from cancer cells, and its potential utility to act as a biomarker for detection of PHCCA, remain largely unclear.

In this study, exosomal Cripto-1 was quantitatively determined in a large number of patient serum samples by an exosome enzyme linked immunosorbent assay (exoELISA) method that was developed herein. The serum samples were collected from healthy control (HC) individuals, cholangitis patients and PHCCA patients. Furthermore, by analyzing a cohort of PHCAA patients with matched sera and tissues samples, we explored whether exosomal Cripto-1 in sera could be a surrogate of tissue biopsy. Finally, this study sought to assess the significance of Cripto-1 in prognosis in a retrospective cohort of PHCCA patients.

## Materials and Methods

### Cell Culture

Human PHCAA cells, QBC-939 and FRH-0201 were purchased from the Cell Bank of the Chinese Academy of Sciences (Shanghai, China) and maintained in Dulbecco’s Modified Eagle medium supplemented with 10% exosome-depleted fetal bovine serum in a humidified atmosphere containing 5% CO_2_.

### Tumor Xenograft Models

Four-week-old BALB/c nude mice were purchased from GemPharmatech (Nanjing, China). A total of 3×10^6^ QBC939 cells transfected with an empty vector or the Cripto-1-OE vector, were subcutaneously injected into the flank of nude mice (24 mice for each group). Tumor diameters were measured every 2 days, and tumor volume was calculated as follows: volume= (length×width^2^)/2. After 2, 4 and 6 weeks, the mice were sacrificed, and their blood and solid tumors were collected. All procedures were approved by the Animal Management Committee of Shandong University.

### Patients and Samples

We obtained the approval of the local ethical committees, and all subjects gave written informed consent to participate. Three cohorts of subjects from Qilu Hospital of Shandong University and Shandong Provincial Third Hospital were enrolled in this study. In the first cohort, pre-operation serum samples were collected from a group of 217 cases with HC (n=65), cholangitis (n=47) and PHCCA (n=115). The PHCCA cases were newly diagnosed and previously untreated; their tumors were confirmed by postoperative histopathological analyses. In the second cohort, sera and matched tissue was collected from 34 PHCCA patients. Moreover, postoperative sera were collected from 15 of the PHCCA cases in the second cohort. The third cohort was comprised of 105 PHCCA patients with available formalin-fixed tumor tissues. These patients underwent radical resection and were followed up regularly. PHCCA was staged according to the 7th AJCC/UICC tumor-node-metastasis (TNM) classification system.

Serum was separated by 2-step centrifugation (1,600g for 10 minutes followed by 16,000g for 10 minutes as previously described (22). Tissues were embedded in paraffin and cut into 4μm sections. For tissue microarray (TMA) construction, core tissues of 1.5 mm in diameter from each tumor block, as confirmed by hematoxylin and eosin staining, were re-embedded into the recipient TMA block as we described previously (2).

### Exosome Isolation and Identification

Exosomes were isolated from conditioned medium (CM) and serum using ultracentrifugation. CM was collected with centrifugation of 800g for 10 min and 2,000g for 10 min. Next, the CM supernatant, or cell-free serum diluted with D-PBS, was filtered through a 0.22μm filter and then ultracentrifuged at 120,000g for 70 min at 4°C. The pellets were washed with D-PBS and purified by further ultracentrifugation at 120,000 g for 70 min at 4°C. Exosomal markers CD81, TSG101 and Calnexin were detected by western blotting assay.

### Western Blotting

Cellular and exosomal lysates were prepared using RIPA buffer (Life Technologies) and quantified using a Pierce™ BCA Protein Assay Kit (Thermo Scientific). Proteins were separated by SDS-PAGE and transferred to PVDF membranes (Millipore). After blocking in 5% skimmed milk for 30 min, the membrane was incubated with primary antibodies overnight at 4°C. The following primary antibodies were used: CD81 (Abcam, ab79559, 1:1000 dilution), TSG101 (Abcam, ab83, 1:1000 dilution), Calnexin(Abcam,ab133615, 1:1000 dilution) and Cripto1 (Abcam, ab108391, 1:1000 dilution). Afterwards, an HRP-conjugated secondary antibody (Beyotime, Shanghai, China) was added at 1:5000 before incubation for 1h at room temperature. Chemiluminescence signal was detected using enhanced ECL Reagent (Vazyme, Nanjing, China) on the FluorChem E Chemiluminescent System (Cell Biosciences, Santa Clara, CA, USA).

### ELISA Detection of Exosomal Cripto-1

The levels of exosomal Cripto-1 were detected using a Human Cripto-1 DuoSet ELISA kit (R&D) with some methodological alterations. In brief, 96 well-plates were coated with 100 μl of anti-CD81antibody (Abcam, ab79559, 1:250 dilution) per well, instead of the Capture Antibody in the kit, before incubation overnight at room temperature. After blocking plates with 300 μl/well Reagent Diluent for at least 1 h, 100 μl exosomes were added and incubated for 2 h at room temperature. Subsequently, we added 100 μl of the Detection Antibody before incubation for 2h, 100μl Streptavidin-HRP for 20 min, and then 100 μl Substrate Solution for 20 min. After blocking with 50 μl Stop Solution, the optical density (OD) of each well was determine immediately at 450 nm on an ELISA plate reader (Thermo Scientific, United States).

### Carbohydrate Antigen 19-9 (CA19-9) and Carcinoembryonic Antigen (CEA) Assays

Serum CEA and CA19-9 levels were detected using an electrochemiluminescence method on the Cobas E601 Analyzer (Roche Diagnostics GmbH, Germany); the upper limits were defined as 5 ng/ml and 37 U/ml according to the manufacturer’s directions.

### Immunohistochemistry (IHC)

Slides were immersed in 3% hydrogen peroxide to inactivate endogenous peroxidase and incubated in EDTA buffer (pH = 9.0) for antigen retrieval. After blocking by 1% bovine serum albumin, sections were incubated with primary antibodies overnight at 4°C. Subsequently, the slides were incubated in secondary antibody prior to 3,3’-diaminobenzidine solution (Zsbio, Beijing, China), which enable visualization. The following primary antibodies were used: Cripto-1 Antibody (R&D, MAB2772, 1:100 dilution), Ki-67 (CST, #9027, 1:400 dilution), E-Cadherin (CST, #3195, 1:400 dilution). The IHC results were evaluated using the Quant Center software and calculated by a histochemistry score (H-score) system (23). The formula for the H-score was: H-score=(percentage of cells of weak intensity×1)+(percentage of cells of moderate intensity×2)+percentage of cells of strong intensity×3).

### Statistical Analysis

A Kruskal-Wallis test was employed for global comparison of Cripto-1, CEA or CA19-9 level among multiple groups, and further *post hoc* multiple comparisons were examined using a Mann–Whitney U test. A chi-square test was used for comparison of categorical variables. Logistic regression modelling was performed to combine biomarkers and generated predicted probability value. The correlation between Cripto-1 and E-cadherin expression was assessed by spearman correlation analysis. Survival curves were generated using the Kaplan-Meier method, and compared by a log-rank test. A Cox regression model was applied for identifying the independent prognostic factors. All above statistical analyses were performed by SPSS software, 22.0 and figures were prepared using GraphPad Prism, 9.1. The area under the curve (AUC) was calculated on the receiver operating characteristic (ROC) curve and compared using MedCalc 9.3.9.0. The cutoff point was determined based on the Youden index (sensitivity+specificity-1). Statistical significance was defined as two-sided *P*<0.05.

## Results

### Cripto-1 Is Secreted With Exosomes From PHCCA Cells

**Figure 1:**
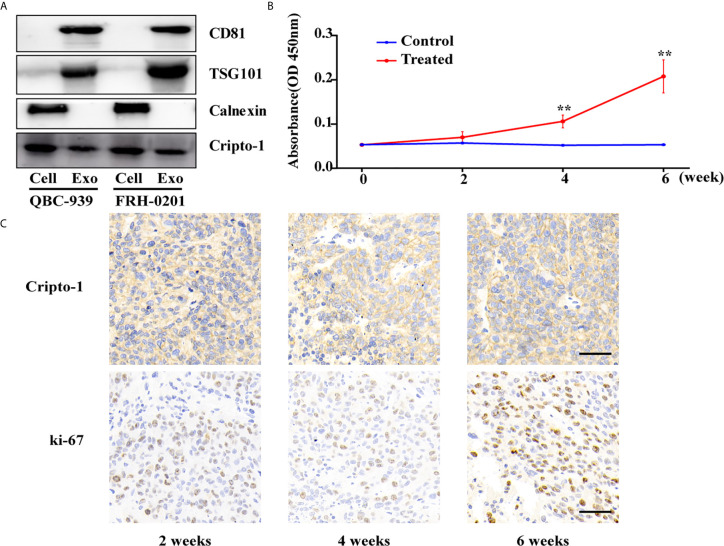
Cripto-1 is secreted with exosomes from PHCCA cells. **(A)** Western blotting analysis of the exosomal markers (CD81, TSG101 and Calnexin) and Cripto-1 in PHCCA cells and exosomes isolated from CM of cells. **(B)** ELISA analysis of exosomal Cripto-1 purified from serum in BALB/c athymic nude mice (control) or mice subcutaneously injected with QBC-939 cells (treated). The levels of exosomal Cripto-1 at 4 week and 6 week in treated group were significantly higher than those in control group and at 0 week in treated group. ^**^ *P* < 0.01(student’s t-test). Data are presented as mean ± standard deviation. **(C)** Xenograft tumor and the corresponding immunohistochemistry of Cripto-1 and Ki-67 at 4 week and 6 week in treated group. Scale bars, 50μm.

We isolated exosomes from the CM of two sets of PHCAA cells, QBC-939 and FRH-0201. Exosomes purified from PHCAA cells showed enrichment of exosomal markers, CD81 and TSG101, compared to total cell lysis, and had no Calnexin expression, an endoplasmic reticulum marker known to be absent in exosomes and present in cells lysis (**Figure 1A**). Western blotting analysis also showed Cripto-1 was expressed both in PHCAA cells and their exosomes (**Figure 1A**).

To observe whether Cripto-1 was released with exosomes *in vivo*, we subcutaneously injected QBC-939 cells into nude mice to establish a PHCCA mouse model. After 2 weeks, 4 weeks and 6 weeks post-injection the mice were bled, and tumors were harvested. As shown in **Figure 1B**, the concentration of serum exosomal Cripto-1 was gradually increased in mice xenografted QBC-939 cells (treated group), but not detected in normal mice (control group). Levels of exosomal Cripto-1 at 4 weeks and 6 weeks in the treated group were significantly higher than those in the control group and at 0 weeks in the treated group. IHC analysis showed the xenograft tumors had high Cripto-1 and ki-67 expression (**Figure 1C**), confirming exosomal Cripto-1 come from injected QBC-939 cells.

### Evaluation of exoELISA Method for Exosomal Cripto-1 Detection

**Figure 2:**
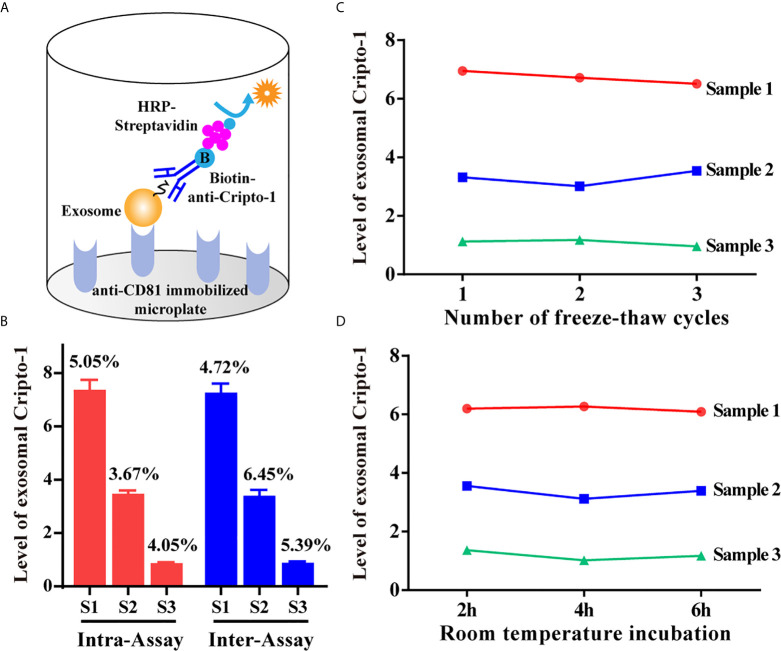
Evaluation of exoELISA method for exosomal Cripto-1 detection. **(A)** Schematic representation of the established exoELISA set for exosomal Cripto-1 detection; **(B)** Coefficient of variation for the exoELISA; **(C)** Serum samples under re-peated freezing and thawing; **(D)** Serum samples incubating at room temperature for different time.

An exoELISA method was developed for detection of exosomal Cripto-1 in serum as shown in **Figure 2A**. Repeat measurements of 3 serum samples, from the same batch or different batches, resulted in an average intra-assay coefficient of variation (CV) and inter-assay CV of 4.27% and 5.52% (**Figure 2B**). Then, the serum samples were evaluated following repeated freezing and thawing, with different lengths of room temperature incubation; the results of this experiment showed no significant changes to the levels of exosomal Cripto-1 (**Figures 2C, D**).

### Levels of Exosomal Cripto-1 Were Increased in PHCCA Patients’ Sera

**Figure 3:**
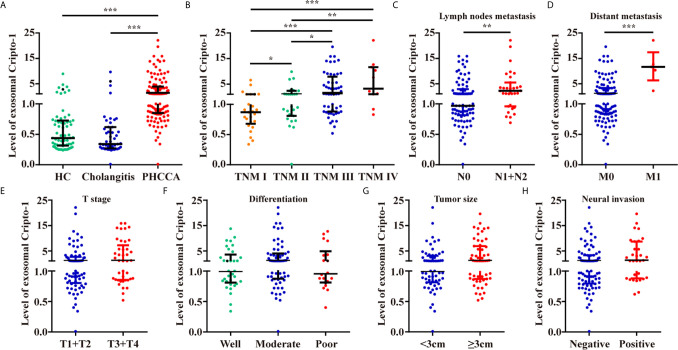
Levels of exosomal Cripto-1 in PHCCA patient sera. **(A)** Levels of exosomal Cripto-1 were compared in sera among healthy control (HC) individuals, cholangitis patients and PHCCA patients. Comparison analysis of exosomal Cripto-1 between different TNM stage **(B)**, lymph nodes metastasis **(C)**, distant metastasis **(D)**, T stage **(E)**, differentiation **(F)**, tumor size **(G)**, neural invasion **(H)** in PHCCA patients. ^*^ *P* < 0.05, ^**^ *P* < 0.01, ^***^ *P* < 0.001(Mann–Whitney U test). Data represents the median (interquartile range).

By analyzing a large number of serum samples, measuring levels of exosomal Cripto-1 in serum showed a significant difference among HC, cholangitis and PHCCA groups (**Table 1**). Moreover, Cripto-1 levels were independent of age and sex (**Table 1**). Further pairwise comparison demonstrated exosomal Cripto-1 was significantly increased in the sera of the PHCCA group compared with the HC and cholangitis groups (both at *P*<0.001), whilst there was no significant difference between the HC and cholangitis groups (**Figure 3A**). In PHCCA patients, the levels of exosomal Cripto-1 increased as the TMN stage increased (**Figure 3B**). Further analysis showed exosomal Cripto-1 levels were significantly elevated in patients with lymph nodes metastasis (**Figure 3C**) or distant metastasis (**Figure 3D**), but not an advanced T stage (**Figure 3E**). Meanwhile, there were no significant differences observed when PHCCA cases were stratified by differentiation (**Figure 3F**), tumor size (**Figure 3G**) and neural invasion (**Figure 3H**). Cripto-1 showed no obvious association with age and sex (**Table S1**). Correlations between levels of exosomal Cripto-1 and clinicopathological characteristics are shown in **Table S1**.

**Table 1:** Characteristics and levels of biomarker of subjects.

	Healthy control	Cholangitis	PHCCA
Cases	65	47	115
Gender(Male/Female)	40/25	31/16	73/42
Age(y) ^a^	59.4 ± 14.5	57.6 ± 14.9	61.3 ± 9.7
CEA (ng/ml) ^bc^	2.78 (1.64-3.80)	2.63 (1.09-3.40)	3.12 (1.65-4.60)
CA19-9 (U/ml) ^bc^	22.1 (13.3-31.0)	17.7 (7.98-23.3)	103 (22.1-441)
Cripto-1(pg/10mg) ^bc^	0.44 (0.32-0.73)	0.34 (0.27-0.62)	1.03 (0.85-3.89)

a Data are presented as mean ± standard deviation.

b Data are presented as median (interquartile range).

c Date are compared using Kruskal-Wallis test, P < 0.001.

### Diagnosis Performance of Serum Exosomal Cripto-1 for PHCCA Patients

**Figure 4:**
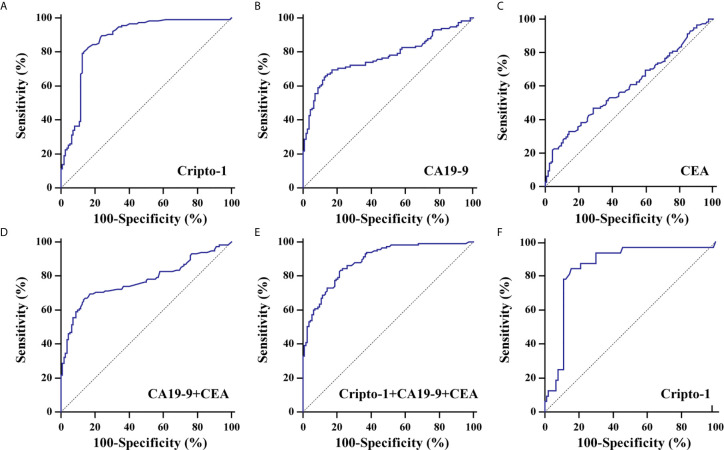
Diagnostic significance analysis of exosomal Cripto-1 and traditional serum markers. ROC curves analysis for the detection of PHCCA using exosomal Cripto-1 **(A)**, CA19-9 **(B)**, CEA **(C)**, combined CA19-9 and CEA **(D)**, and combined exosomal Cripto-1, CA19-9 and CEA **(E)** in all subjects. **(F)** ROC curves analysis for the detection of PHCCA using exosomal Cripto-1 in both CA19-9 and CEA negative individuals.

ROC curve analyses realized that exosomal Cripto-1 was robust in discerning PHCCA patients (n=115) from cholangitis (n=47) and HC subjects (n=65), with an AUC of 0.87 4 (**Figure 4A**). When the Youden index reached a maximum from the ROC curve, the corresponding cutoff value of exosomal Cripto-1 for diagnosing PHCCA was 0.82, achieving a sensitivity of 79.1% and a specificity of 87.5%. Moreover, it was highly capable in discerning different TNM stage of PHCCA patients from cholangitis and HC subjects (**Figure S1**). In contrast, exosomal Cripto-1 levels could not distinguish cholangitis cases from healthy controls (**Figure S2**). And, exosomal Cripto-1 had limited differential diagnosis value for PHCCA with or without lymph nodes metastasis (**Figure S3**).

To better understand the diagnostic performance of exosomal Cripto-1 for PHCCA, the levels of traditional serum tumor markers, CA19-9 and CEA, were assessed in all subjects. As shown in **Table 1**, there were significant differences between the HC, cholangitis and PHCCA groups. Further, CA19-9 in the PHCCA group was increased compared to the cholangitis and HC groups (both at *P*< 0.001), while CEA was only significantly higher than patients in the cholangitis group (*P*< 0.01) (**Figure S4**). The AUC values of CA19-9, CEA and the combined CA19-9 and CEA for PHCCA diagnosis were 0.773 (**Figure 4B**) and 0.596 (**Figure 4C**) and 0.773 (**Figure 4D**), much lower than that of exosomal Cripto-1. When the combined exosomal Cripto-1, CA19-9 and CEA levels were used, the AUC was 0.888(**Figure 4E**), not significantly enhanced compared to exosomal Cripto-1 alone. Since exosomal Cripto-1 had no significant association with CA19-9 and CEA, and no significant difference was observed between CA19-9 or CEA positive and negative groups (**Figure S5**), its diagnosis performance was assessed for CA19-9 or CEA negative individuals. As shown in **Figure 4F**, the exosomal Cripto-1 also had a high diagnostic capacity in subjects where diagnosis was missed in clinics. The diagnostic performance characteristics of exosomal Cripto-1, CA19-9 and CEA for PHCCA, such as sensitivity, specificity, positive likelihood ratio and negative likelihood ratio, are shown in **Table 2**.

**Table 2:** The diagnosis performance of Cripto-1, CA19-9 and CEA for PHCCA.

Index	AUC	95% CI	Sensitivity	Specificity	+LR	-LR
Cripto-1	0.874	0.824-0.914	79.1	87.5	6.33	0.24
CA199	0.773	0.713-0.826	65.2	87.5	5.22	0.40
CEA	0.596	0.529-0.660	33.0	85.7	2.31	0.78
CA199+CEA	0.773	0.713-0.825	66.1	86.6	4.93	0.39
Cripto-1+CA199+CEA	0.888	0.840-0.926	82.6	78.6	3.86	0.22

AUC, Area under curve; CI, Confidence interval; +LR, Positive likelihood ratio; -LR, Negative likelihood ratio.

### Levels of Exosomal Cripto-1 in Sera of PHCAA Patients Reflects Their Expression in Tissues

**Figure 5:**
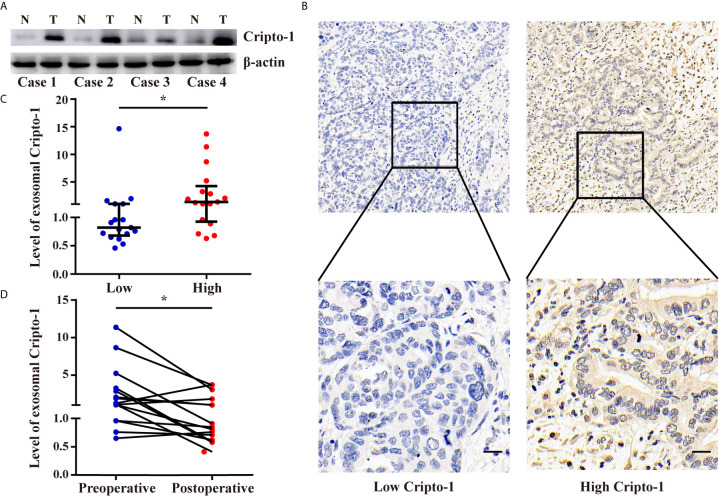
Levels of exosomal Cripto-1 in sera of PHCAA patients reflects their expression in tissues. **(A)** Western blotting analysis of Cripto-1 expression in PHCCA tissues and adjacent normal tissues. **(B)** Immunohistochemistry analysis of Cripto-1 expression in PHCCA tissues. PHCCA patients were classified into low and high Cripto-1 expression groups according to median of histochemistry score (H-score). Scale bars, 50μm **(C)** exoELISA was performed to compare the levels of exosomal Cripto-1 in sera between PHCAA patients with low and high Cripto-1 expression in tissues. ^*^ *P* < 0.05 (Mann–Whitney U test). Data represents the median (interquartile range). **(D)** Preoperative and perioperative levels of exosomal Cripto-1 in sera were compared in PHCAA patients. ^*^ *P* < 0.05 (Wilcoxon matched-pairs signed rank test).

To explore the relationship between Cripto-1 in the exosomes of sera and tissues, 34 pairs of tumor tissues and sera were collected from PHCCA patients who underwent radical resection. Western blot analysis showed Cripto-1 was increased in PHCCA tissues compared to the corresponding normal bile duct tissues (**Figure 5A**). Then, the expression of Cripto-1 in 34 PHCCA tissue samples was detected by immunohistochemical staining and classified into low and high Cripto-1 expression groups, according to median of H-score (**Figure 5B**). The corresponding sera detected by exoEILSA showed that exosomal Cripto-1 levels in immunohistochemically Cripto-1-high cases, were significantly higher than in Cripto-1-low cases (**Figure 5C**). Among the aforementioned cases, 15 further cases were detected 1-week post surgery. The quantitative assay showed that after surgery, the median levels of exosomal Cripto-1 decreased from 1.25 (0.96-3.26) to 0.85(0.62-1.82) (**Figure 5D**).

### Expression and Prognostic Significance of Cripto-1 in PHCCA Tissues

**Figure 6:**
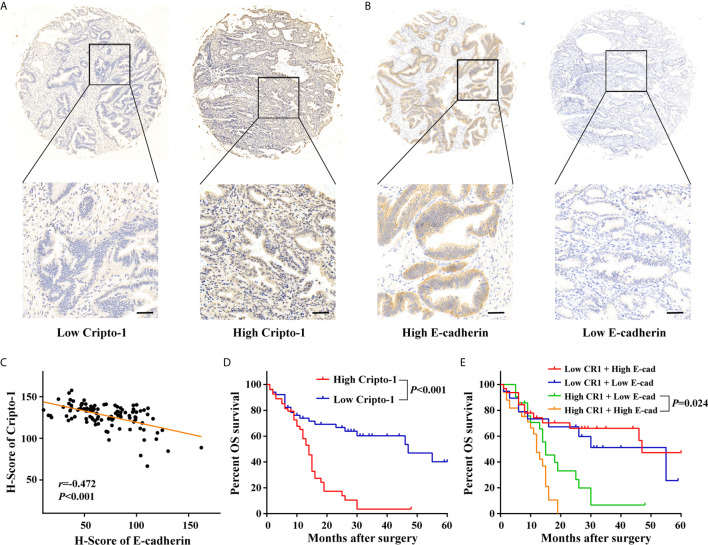
Expression and prognostic significance of Cripto-1 in PHCCA tissues. **(A, B)** Immunohistochemistry analysis of Cripto-1 and E-cadherin expression in PHCCA tissues. PHCCA were classified into low and high Cripto-1/E-cadherin expression groups according to median of H-score. Scale bars, 50μm **(C)** Correlation analyses between Cripto-1 and E-cadherin expression in PHCCA tissues. **(D)** Kaplan–Meier curve for overall survival in PHCCA patients stratified according to Cripto-1 expression. **(E)** Kaplan–Meier curve for overall survival in PHCCA patients stratified according to Cripto-1 expression and E-cadherin expression.

Anti-Cripto-1 was used for immunohistochemical staining, in a retrospective cohort of 105 cases with PHCCA who had underwent radical resection. By evaluating the H-scores, the patients were divided into subgroups with low or high expression of Cripto-1(**Figure 6A**). Data from **Table S2** showed Cripto-1 expression significantly associated with lymph nodes metastasis, whilst there was no relationship with other clinicopathological factors, such as gender, age, differentiation, tumor size, T stage and neural invasion. As patients with high exosomal Cripto-1 expression in tissues or serum tended to have metastasis and EMT was a critical event involved in tumor metastasis (10, 11), the expression of E-cadherin, a known EMT marker, was assessed (**Figure 6B**). As shown in **Figure 6C**, the Cripto-1 expression negatively correlated with E-cadherin expression and patients with high Cripto-1 expression lowly expressed E-cadherin.

To investigate the prognostic properties of PHCCA, univariate analysis was performed using a Kaplan–Meier survival curve. As shown in **Figure 6D**, patients with high Cripto-1 expression had significantly lower survival rates than patients with low Cripto-1 expression. Moreover, patients with high Cripto-1 and low E-cadherin expression had the lowest probability of survival (**Figure 6E**). However, there were no significant survival differences between patients with low and high E-cadherin expression (**Figure S6**). In addition, patients with advanced T stage or positive lymph nodes metastasis had poor overall survival (**Figure S7**). Cripto-1 expression, along with the clinicopathological factors, was subjected to Cox model for multivariate analysis; except for T stage, Cripto-1 was identified as an independent prognostic biomarker (**Table 3**).

**Table 3:** Univariate and multivariate analysis of prognostic factors predicting overall survival in PHCAA patients.

Parameters	Univariate analysis		Multivariate analysis	
	5-year OS rate (%)	*P*-value^a^	HR(95%IC)	*P*-value^b^
Gender (Male *VS* Female)	0 *VS* 31.4	0.417	0.882 (0.491-1.582)	0.673
Age(<65y *VS* ≥65y)	29.1 *VS* 0	0.121	1.567 (0.877-2.800)	0.129
Differentiation(Well *VS* Moderate *VS* Poor)	36.7 *VS* 23.7 *VS* 14.1	0.056	1.373 (0.918-2.053)	0.123
Tumor size(<3cm *VS* ≥3cm)	30.7 *VS* 13.3	0.171	1.455 (0.862-2.455)	0.160
T stage (T1+T2 *VS* T3+T4)	32.1 *VS* 10.3	0.001	1.469 (1.115-1.936)	0.006
Lymph nodes metastasis(N0 *VS* N1+N2)	27.8 *VS* 7.3	0.008	1.396 (0.746-2.614)	0.297
Neural invasion(Negative *VS* Positive)	20.7 *VS* 34.7	0.930	1.341 (0.682-2.639)	0.395
Cripto-1 (Negative *VS* Positive)	40.2 *VS* 3.5	<0.001	2.460 (1.276-4.743)	0.007

OS, Overall survival; HR, Hazard ratio; CI, Confidence interval; ^a^Calculated by log-rank test; ^b^Calculated by Cox-regression Hazard model.

## Discussion

In the current study, it was first reported that Cripto-1 was released *via* exosomes by PHCCA cells. Next, an exoELISA method was developed and 3 cohorts were used to evaluate the clinical application value of Cripto-1. The study has achieved several new results. Firstly, exosomal Cripto-1 were increased in sera of PHCCA patients and associated with metastasis. Compared with traditional serum tumor markers, CA19-9 and CEA, exosomal Cripto-1 demonstrated as a more appropriate marker for PHCCA diagnosis. Secondly, it was found that exosomal Cripto-1 levels in sera reflect the expression in the tissues of PHCAA patients. Thirdly, it has been demonstrated in a prospective cohort analysis that Cripto-1 might be an independent factor for identifying poor prognosis in patients with PHCAA. Together, these findings verify that exosomal Cripto-1 is a potential biomarker for the diagnosis and prognosis of PHCCA.

In recent years, exosomes have emerged as an important liquid biopsy tool for a variety of malignant tumors (24). With the development of mass spectrometry and other detection technologies, the protein composition of exosomes has been constantly identified; some of these are already used as tumor biomarkers due to their stability and specificity (20, 25). Amongst them, Glycosyl-Phosphatidyl-Inositol (GPI) anchored proteins (GPI-APs) are attractive as a source of exosomal candidates (26). This is because GPI-APs are preferentially embedded into lipid rafts through their GPI anchor and the lipid raft microdomains are believed to be involved in exosome biogenesis (27, 28). For example, Glypican-1 has been found in pancreatic carcinoma cell derived exosomes and can serve as a potential non-invasive diagnostic biomarker to facilitate the early detection of pancreatic cancer (29). CD24, located in lipid rafts through its GPI anchor, is demonstrated to be found in high levels in exosomes isolated from ovarian cancer cells, compared to other tumor biomarkers such as CA-125, EGFR and MUC18 (30). Since Cripto-1 has been reported as a cell surface GPI-linked glycoprotein (31), this study tested whether it can be found in exosomes. Cripto-1 was found in exosomes from the culture medium of PHCCA cell lines and the peripheral blood of mice implanted PHCCA cells. Thus, exosomal Cripto-1 might provide a noninvasive biomarker for the detection of PHCCA.

In previous studies, Cripto-1 has been detected at the mRNA or protein levels in tissues of several human solid cancers. Furthermore, Cripto-1 protein levels are also increased in serum/plasma obtained from patients with glioblastoma (32), renal cell carcinoma (33), colon cancer and breast cancer (34). In the present study, Cripto-1 levels were quantitatively determined in the exosomes of serum samples and showed increased levels in PHCCA patients compared to cholangitis patients and healthy controls. Moreover, the results are reliable, with batch differentiation, repeated freeze thawing and different lengths of incubation proving to have no significant effect on the result. To better assess the application of exosomal Cripto-1 as a potential diagnosis marker, the study compared its ability to detect PHCCA with current diagnostic tools CA19-9 and CEA, the traditional serum tumor markers routinely used in clinic. As shown in this data, exosomal Cripto-1 demonstrated improved sensitivity and specificity, associated with a markedly larger AUC, in distinguishing PHCCA from cholangitis patients and healthy controls. Furthermore, exosomal Cripto-1 also had a high diagnostic capacity in CA19-9 and CEA negative individuals. However, combined detection of Cripto-1, CA19-9 and CEA did not enhance diagnostic performance compared to Cripto-1 alone. Another interesting finding of the current study is that exosomal Cripto-1 levels increased as the PHCCA TNM stage progressed. However, this phenomenon was not observed in CA19-9 or CEA. Thus, it can be concluded that exosomal Cripto-1 may detect PHCCA patients at an earlier stage.

Our previous study has reported that several long noncoding RNAs in exosomes of serum are associated their expression in tumor tissues (35). To better understand whether circulating exosomal Cripto-1 is represented in tissue biopsies, Cripto-1 expression was further examined in the tissues of PHCAA patients. Data from this study showed that Cripto-1 levels were increased in PHCCA tissues compared with the corresponding normal bile duct tissues. Exosomal Cripto-1 levels were especially elevated in immunohistochemically Cripto-1-high cases. These results infer that exosomal Cripto-1 levels in patient sera can reflect expression in tissues of PHCCA patients. Moreover, levels of exosomal Cripto-1 were decreased after surgery, which is conducive to monitoring disease status. Due to the limited follow-up time of collected serum, the prognostic value of Cripto-1 was analyzed using a previously established tissue microarray. It was found that patients with high expression of Cripto-1 had poor survival, which was an independent prognostic biomarker. This phenomenon is similar to some studies on other cancers, such as non-small cell lung cancer (36), hepatocellular carcinoma (37) and esophageal squamous cell carcinoma (38).

In the present study, it was demonstrated that Cripto-1 expression in both serum and tissues significantly associated with metastasis. Several studies have showed Cripto-1 plays an important role during developmental EMT. For example, the over-expression of Cripto-1 down-regulates E-cadherin whilst up regulating β-catenin in prostate cancer cells, thus inducing EMT through activation of the Wnt/β-catenin signaling pathway (39). In clear cell renal cell carcinoma, Cripto-1 promoted EMT properties, including the down-regulation of E-cadherin and the up-regulation of Vimentin, N-cadherin, ZEB-1, and Snail (33). EMT has been reported to increase the motility and invasiveness of cholangiocarcinoma cells (40–42). Functional loss of E-cadherin has been considered a hallmark of EMT (43) and this study showed that Cripto-1 expression negatively correlated with E-cadherin expression in PHCCA tissues; this suggests that Cripto-1 may also play a role as an EMT inducer to promote PHCCA cell migration and invasion. Additionally, it has also been shown that the combination of Cripto-1 and E-cadherin improved the power of the survival prediction.

Although the findings of this study are promising, some limitations need to be addressed. Firstly, it was found that there were increased exosomal Cripto-1 levels in some healthy individuals or cholangitis patients. The cause of this is not clear and whether these subjects develop tumors remains to be seen. Secondly, the prognostic value of exosomal Cripto-1 levels in the sera of PHCCA patients needs to be further studied, although it has been shown to correlate with tissue biopsies in this study. Thirdly, since currently there are not enough follow up serum samples for analysis, further ongoing perspective studies are necessary to address whether exosomal Cripto-1 can identify patients with reoccurring tumors. This is of particular importance for PHCCA patients because of the high recurrence rate (44).

In conclusion, this study developed a convenient and sensitive assay to detect exosomal Cripto-1 and for the first time demonstrated exosomal Cripto-1 as a potential noninvasive maker for PHCCA diagnosis and monitoring. Moreover, Cripto-1 shows promise as an independent predictor of poor prognosis for PHCCA patients. Since Cripto-1 has many advantages over conventional cancer markers, further multi-center prospective studies are needed to confirm whether exosomal Cripto-1 can be incorporated into routine clinical practice.

## Data Availability Statement

The raw data supporting the conclusions of this article will be made available by the authors, without undue reservation.

## Ethics Statement

The studies involving human participants were reviewed and approved by Ethics Committee of Qilu Hospital of Shandong University. The patients/participants provided their written informed consent to participate in this study. The animal study was reviewed and approved by Medical Ethics Committee of Qilu Hospital of Shandong University.

## Author Contributions

CH, YZ, and XZhe performed all experiments. CH and MZ participated in data analysis and interpretation of results. CH, TL, and QG drafted the manuscript. XZha designed the study and revised the manuscript. All authors contributed to the article and approved the submitted version.

## Funding

This work was supported by Shandong Province Natural Science Foundation (ZR2020MH238); Shandong Medical and Health Technology Development Project (2018WSB20002) and National Natural Science Foundation of China (No. 81900728).

## Conflict of Interest

The authors declare that the research was conducted in the absence of any commercial or financial relationships that could be construed as a potential conflict of interest.

## Publisher’s Note

All claims expressed in this article are solely those of the authors and do not necessarily represent those of their affiliated organizations, or those of the publisher, the editors and the reviewers. Any product that may be evaluated in this article, or claim that may be made by its manufacturer, is not guaranteed or endorsed by the publisher.

## References

[1] Siriwardena AK. Klatskin Tumor. J Clin Oncol (2017) 35(36):4091–2. 10.1200/JCO.2017.75.1586 28985102

[2] Liu Z Sun R Zhang X Qiu B Chen T Li Z. Transcription Factor 7 Promotes the Progression of Perihilar Cholangiocarcinoma by Inducing the Transcription of c-Myc and FOS-Like Antigen 1. EBioMedicine (2019) 45:181–91. 10.1016/j.ebiom.2019.06.023 PMC6642257 31248836

[3] Xu YF Liu ZL Pan C Yang XQ Ning SL Liu HD. HMGB1 Correlates With Angiogenesis and Poor Prognosis of Perihilar Cholangiocarcinoma *via* Elevating VEGFR2 of Vessel Endothelium. Oncogene (2019) 38(6):868–80. 10.1038/s41388-018-0485-8 30177842

[4] Groot Koerkamp B Wiggers JK Gonen M Doussot A Allen PJ Besselink MGH. Survival After Resection of Perihilar Cholangiocarcinoma-Development and External Validation of a Prognostic Nomogram. Ann Oncol (2015) 26(9):1930–5. 10.1093/annonc/mdv279 PMC4754626 26133967

[5] Nault JC Villanueva A. Biomarkers for Hepatobiliary Cancers. Hepatology (2021) 73 Suppl 1:115–27. 10.1002/hep.31175 32045030

[6] Tsukahara T Ebata T Shimoyama Y Yokoyama Y Igami T Sugawara G. Residual Carcinoma *In Situ* at the Ductal Stump has a Negative Survival Effect: An Analysis of Early-Stage Cholangiocarcinomas. Ann Surg (2017) 266(1):126–32. 10.1097/SLA.0000000000001944 27501166

[7] Watanabe K Nagaoka T Lee JM Bianco C Gonzales M Castro NP. Enhancement of Notch Receptor Maturation and Signaling Sensitivity by Cripto-1. J Cell Biol (2009) 187(3):343–53. 10.1083/jcb.200905105 PMC2779239 19948478

[8] Park SW Do HJ Han MH Choi W Kim JH. The Expression of the Embryonic Gene Cripto-1 Is Regulated by OCT4 in Human Embryonal Carcinoma NCCIT Cells. FEBS Lett (2018) 592(1):24–35. 10.1002/1873-3468.12935 29223130

[9] Klauzinska M Castro NP Rangel MC Spike BT Gray PC Bertolette D. The Multifaceted Role of the Embryonic Gene Cripto-1 in Cancer, Stem Cells and Epithelial-Mesenchymal Transition. Semin Cancer Biol (2014) 29:51–8. 10.1016/j.semcancer.2014.08.003 PMC4252566 25153355

[10] Rangel MC Karasawa H Castro NP Nagaoka T Salomon DS Bianco C. Role of Cripto-1 During Epithelial-to-Mesenchymal Transition in Development and Cancer. Am J Pathol (2012) 180(6):2188–200. 10.1016/j.ajpath.2012.02.031 PMC3378914 22542493

[11] Pilli VS Gupta K Kotha BP Aradhyam GK. Snail-Mediated Cripto-1 Repression Regulates the Cell Cycle and Epithelial-Mesenchymal Transition-Related Gene Expression. FEBS Lett (2015) 589(11):1249–56. 10.1016/j.febslet.2015.04.005 25889638

[12] Bianco C Strizzi L Rehman A Normanno N Wechselberger C Sun Y. A Nodal- and ALK4-Independent Signaling Pathway Activated by Cripto-1 Through Glypican-1 and C-Src. Cancer Res (2003) 63(6):1192–7. 12649175

[13] Bianco C Strizzi L Ebert A Chang C Rehman A Normanno N. Role of Human Cripto-1 in Tumor Angiogenesis. J Natl Cancer Inst (2005) 97(2):132–41. 10.1093/jnci/dji011 15657343

[14] Ishii H Zahra MH Takayanagi A Seno M. A Novel Artificially Humanized Anti-Cripto-1 Antibody Suppressing Cancer Cell Growth. Int J Mol Sci (2021) 22(4):1709. 10.3390/ijms22041709 33567764 PMC7915030

[15] Sun G Yan SS Shi L Wan ZQ Jiang N Fu LS. MicroRNA-15b Suppresses the Growth and Invasion of Glioma Cells Through Targeted Inhibition of Cripto-1 Expression. Mol Med Rep (2016) 13(6):4897–903. 10.3892/mmr.2016.5126 27082313

[16] Soung YH Ford S Zhang V Chung J. Exosomes in Cancer Diagnostics. Cancers (Basel) (2017) 9(1):94–100. 10.3390/cancers9010008 PMC5295779 28085080

[17] Sung BH von Lersner A Guerrero J Krystofiak ES Inman D Pelletier R. A Live Cell Reporter of Exosome Secretion and Uptake Reveals Pathfinding Behavior of Migrating Cells. Nat Commun (2020) 11(1):2092. 10.1038/s41467-020-15747-2 32350252 PMC7190671

[18] Li W Li C Zhou T Liu X Liu X Li X. Role of Exosomal Proteins in Cancer Diagnosis. Mol Cancer (2017) 16(1):145. 10.1186/s12943-017-0706-8 28851367 PMC5576100

[19] Kalluri R. The Biology and Function of Exosomes in Cancer. J Clin Invest (2016) 126(4):1208–15. 10.1172/JCI81135 PMC4811149 27035812

[20] Hoshino A Kim HS Bojmar L Gyan KE Cioffi M Hernandez J. Extracellular Vesicle and Particle Biomarkers Define Multiple Human Cancers. Cell (2020) 182(4):1044–61.e18. 10.1016/j.cell.2020.07.009 32795414 PMC7522766

[21] Kimura H Yamamoto H Harada T Fumoto K Osugi Y Sada R. CKAP4, a DKK1 Receptor, Is a Biomarker in Exosomes Derived From Pancreatic Cancer and a Molecular Target for Therapy. Clin Cancer Res (2019) 25(6):1936–47. 10.1158/1078-0432.CCR-18-2124 30610103

[22] Zhang X Yang X Zhang Y Liu X Zheng G Yang Y. Direct Serum Assay for Cell-Free Bmi-1 mRNA and Its Potential Diagnostic and Prognostic Value for Colorectal Cancer. Clin Cancer Res (2015) 21(5):1225–33. 10.1158/1078-0432.CCR-14-1761 25547677

[23] Budwit-Novotny DA McCarty KS Cox EB Soper JT Mutch DG Creasman WT. Immunohistochemical Analyses of Estrogen Receptor in Endometrial Adenocarcinoma Using a Monoclonal Antibody. Cancer Res (1986) 46(10):5419–25. 3756890

[24] Li S Yi M Dong B Tan X Luo S Wu K. The Role of Exosomes in Liquid Biopsy for Cancer Diagnosis and Prognosis Prediction. Int J Cancer (2021) 148(11):2640–51. 10.1002/ijc.33386 PMC8049049 33180334

[25] Pietrowska M Zebrowska A Gawin M Marczak L Sharma P Mondal S. Proteomic Profile of Melanoma Cell-Derived Small Extracellular Vesicles in Patients’ Plasma: A Potential Correlate of Melanoma Progression. J Extracell Vesicles (2021) 10(4):e12063. 10.1002/jev2.12063 33613873 PMC7876545

[26] Muller GA. The Release of Glycosylphosphatidylinositol-Anchored Proteins From the Cell Surface. Arch Biochem Biophys (2018) 656:1–18. 10.1016/j.abb.2018.08.009 30120921

[27] Vidal M. Exosomes and GPI-Anchored Proteins: Judicious Pairs for Investigating Biomarkers From Body Fluids. Adv Drug Delivery Rev (2020) 161-162:110–23. 10.1016/j.addr.2020.08.006 32828789

[28] Tan SS Yin Y Lee T Lai RC Yeo RW Zhang B. Therapeutic MSC Exosomes Are Derived From Lipid Raft Microdomains in the Plasma Membrane. J Extracell Vesicles (2013) 2:22614. 10.3402/jev.v2i0.22614 PMC3873122 24371518

[29] Melo SA Luecke LB Kahlert C Fernandez AF Gammon ST Kaye J. Glypican-1 Identifies Cancer Exosomes and Detects Early Pancreatic Cancer. Nature (2015) 523(7559):177–82. 10.1038/nature14581 PMC4825698 26106858

[30] Im H Shao H Park YI Peterson VM Castro CM Weissleder R. Label-Free Detection and Molecular Profiling of Exosomes With a Nano-Plasmonic Sensor. Nat Biotechnol (2014) 32(5):490–5. 10.1038/nbt.2886 PMC4356947 24752081

[31] Alam MJ Takahashi R Afify SM Oo AKK Kumon K Nawara HM. Exogenous Cripto-1 Suppresses Self-Renewal of Cancer Stem Cell Model. Int J Mol Sci (2018) 19(11):3345. 10.3390/ijms19113345 PMC6274844 30373174

[32] Pilgaard L Mortensen JH Henriksen M Olesen P Sorensen P Laursen R. Cripto-1 Expression in Glioblastoma Multiforme. Brain Pathol (2014) 24(4):360–70. 10.1111/bpa.12131 PMC8029290 24521322

[33] Xue YJ Chen SN Chen WG Wu GQ Liao YF Xu JB. Cripto-1 Expression in Patients With Clear Cell Renal Cell Carcinoma Is Associated With Poor Disease Outcome. J Exp Clin Cancer Res (2019) 38(1):378. 10.1186/s13046-019-1386-6 31455359 PMC6712621

[34] Bianco C Strizzi L Mancino M Rehman A Hamada S Watanabe K. Identification of Cripto-1 as a Novel Serologic Marker for Breast and Colon Cancer. Clin Cancer Res (2006) 12(17):5158–64. 10.1158/1078-0432.CCR-06-0274 16951234

[35] Zhang Y Liu H Liu X Guo Y Wang Y Dai Y. Identification of an Exosomal Long Non-Coding RNAs Panel for Predicting Recurrence Risk in Patients With Colorectal Cancer. Aging (Albany NY) (2020) 12(7):6067–88. 10.18632/aging.103006 PMC7185113 32246818

[36] Xu C Yuan Q Hu H Wang W Zhang Q Li L. Expression of Cripto-1 Predicts Poor Prognosis in Stage I Non-Small Cell Lung Cancer. J Cell Mol Med (2020) 24(17):9705–11. 10.1111/jcmm.15518 PMC7520286 32697011

[37] Huang T Guo YZ Yue X Zhang GP Zhang Y Kuang M. Cripto-1 Promotes Tumor Invasion and Predicts Poor Outcomes in Hepatocellular Carcinoma. Carcinogenesis (2020) 41(5):571–81. 10.1093/carcin/bgz133 32649753

[38] Liu Q Cui X Yu X Bian BS Qian F Hu XG. Cripto-1 Acts as a Functional Marker of Cancer Stem-Like Cells and Predicts Prognosis of the Patients in Esophageal Squamous Cell Carcinoma. Mol Cancer (2017) 16(1):81. 10.1186/s12943-017-0650-7 28431580 PMC5399850

[39] Liu Y Qin Z Yang K Liu R Xu Y. Cripto-1 Promotes Epithelial-Mesenchymal Transition in Prostate Cancer *via* Wnt/Beta-Catenin Signaling. Oncol Rep (2017) 37(3):1521–8. 10.3892/or.2017.5378 28098905

[40] Chen T Li K Liu Z Liu J Wang Y Sun R. WDR5 Facilitates EMT and Metastasis of CCA by Increasing HIF-1alpha Accumulation in Myc-Dependent and Independent Pathways. Mol Ther (2021) 29(6):2134–50. 10.1016/j.ymthe.2021.02.017 PMC8178527 33601056

[41] Li Z Liu J Chen T Sun R Liu Z Qiu B. HMGA1-TRIP13 Axis Promotes Stemness and Epithelial Mesenchymal Transition of Perihilar Cholangiocarcinoma in a Positive Feedback Loop Dependent on C-Myc. J Exp Clin Cancer Res (2021) 40(1):86. 10.1186/s13046-021-01890-1 33648560 PMC7923631

[42] Sun R Liu Z Qiu B Chen T Li Z Zhang X. Annexin10 Promotes Extrahepatic Cholangiocarcinoma Metastasis by Facilitating EMT *via* PLA2G4A/PGE2/STAT3 Pathway. EBioMedicine (2019) 47:142–55. 10.1016/j.ebiom.2019.08.062 PMC6796529 31492557

[43] Loh CY Chai JY Tang TF Wong WF Sethi G Shanmugam MK. The E-Cadherin and N-Cadherin Switch in Epithelial-to-Mesenchymal Transition: Signaling, Therapeutic Implications, and Challenges. Cells (2019) 8(10):1118. 10.3390/cells8101118 PMC6830116 31547193

[44] Nakahashi K Ebata T Yokoyama Y Igami T Mizuno T Yamaguchi J. How Long Should Follow-Up be Continued After R0 Resection of Perihilar Cholangiocarcinoma? Surgery (2020) 168(4):617–24. 10.1016/j.surg.2020.04.068 32665142

