# Supplementary material for: Exosomal Cripto-1 Serves as a Potential Biomarker for Perihilar Cholangiocarcinoma
Source: Front Oncol. 2021 Aug 9;11:730615. doi: 10.3389/fonc.2021.730615 (PMC8380828; doi:10.3389/fonc.2021.730615)
Supplement: Supplementary file 1 [file DataSheet_1.zip › supplementary materials/Supplementary Figure legend.docx]

**Supplemental figure legend**

**Figure S1.** Diagnostic performance of exosomal Cripto-1 for different TNM stage of PHCCA patients. ROC curves analysis for distinguishing TNM I (A), TNM II (B), TNM III (C), TNM IV (D) from cholangitis and healthy controls using exosomal Cripto-1.

**Figure S2.** Diagnostic performance of exosomal Cripto-1 and traditional serum markers. ROC curves analysis for distinguishing cholangitis from healthy controls using exosomal Cripto-1(A), CA19-9 (B), and CEA (C).

**Figure S3.** Diagnostic performance of exosomal Cripto-1 for distinguishing PHCCA with lymph nodes metastasis from those without lymph nodes metastasis.

**Figure S4.** Levels of CA19-9 (A) and CEA (B) were compared in sera among healthy control individuals, cholangitis patients and PHCCA patients. ^**^*P*<0.01, ^***^*P*<0.001(Mann–Whitney U test). Data represents the median (interquartile range).

**Figure S5.** The relationship between exosomal Cripto-1 and traditional serum markers. (A) The correlation analysis between exosomal Cripto-1 and CA19-9; (B) The correlation analysis between exosomal Cripto-1 and CEA; (C) Levels of exosomal Cripto-1 between CA19-9 positive and negative groups; (D) Levels of exosomal Cripto-1 between CEA positive and negative groups.

**Figure S6.** Kaplan–Meier curve for overall survival in PHCCA patients stratified according to E-cadherin expression.

**Figure S7.** Kaplan–Meier curve for overall survival in PHCCA patients stratified according to T stage (A) and lymph nodes metastasis (B).
