# Supplementary figures and images for: Exosomal Cripto-1 Serves as a Potential Biomarker for Perihilar Cholangiocarcinoma
Source: Front Oncol. 2021 Aug 9;11:730615. doi: 10.3389/fonc.2021.730615 (PMC8380828; doi:10.3389/fonc.2021.730615)

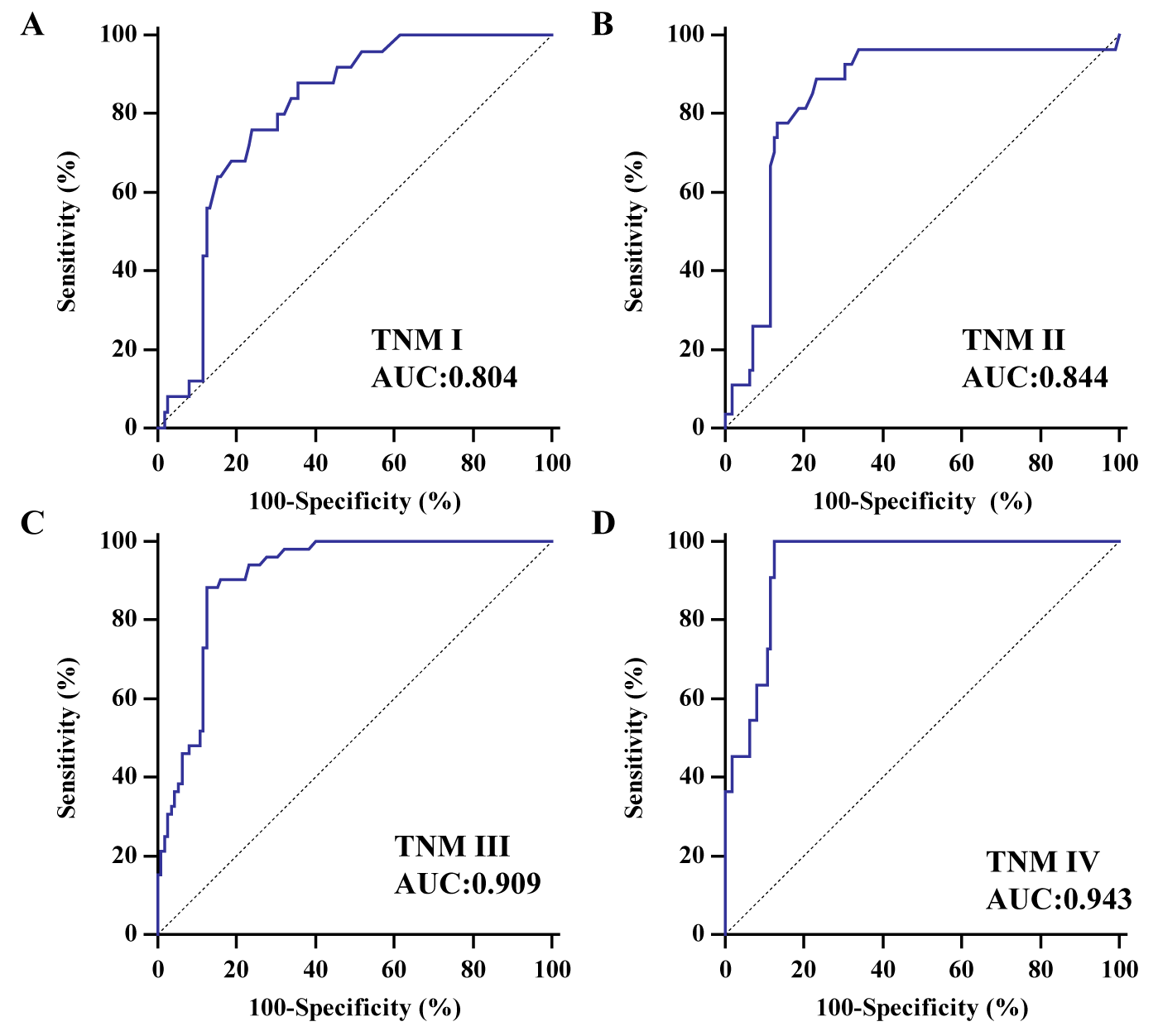

Supplement: Supplementary file 1 [file DataSheet_1.zip › supplementary materials/figure s1.tif]

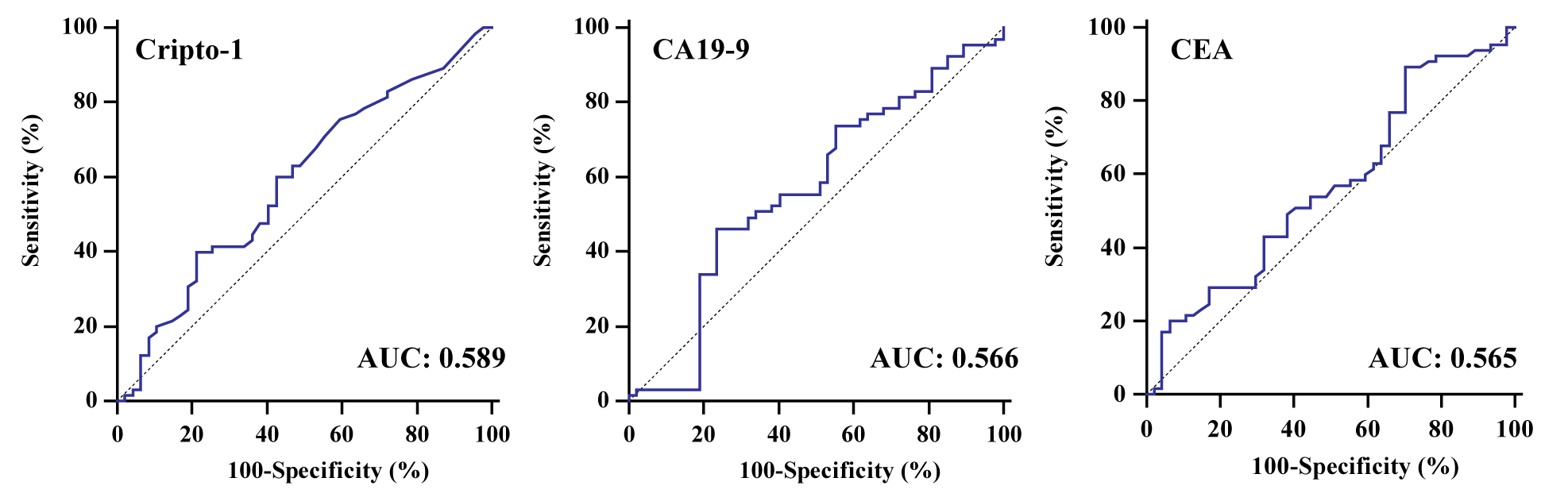

Supplement: Supplementary file 1 [file DataSheet_1.zip › supplementary materials/figure s2.tif]

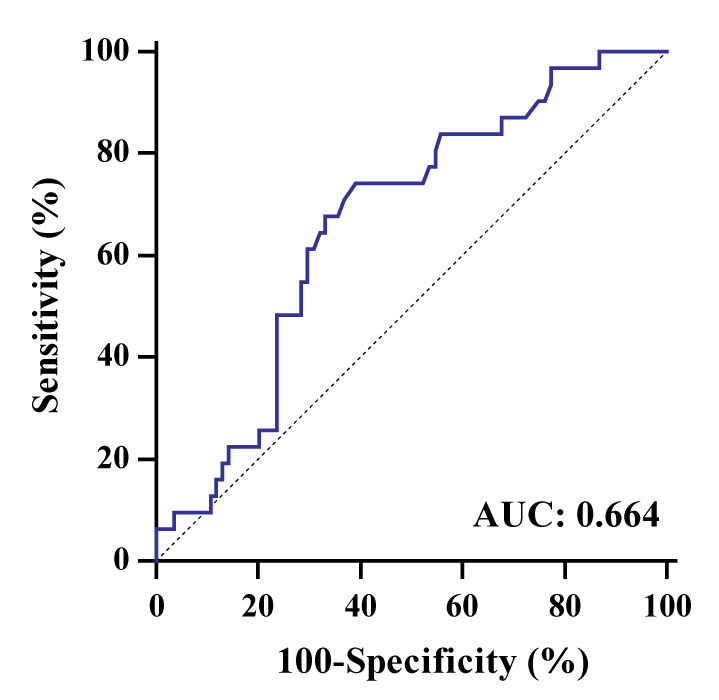

Supplement: Supplementary file 1 [file DataSheet_1.zip › supplementary materials/figure s3.tif]

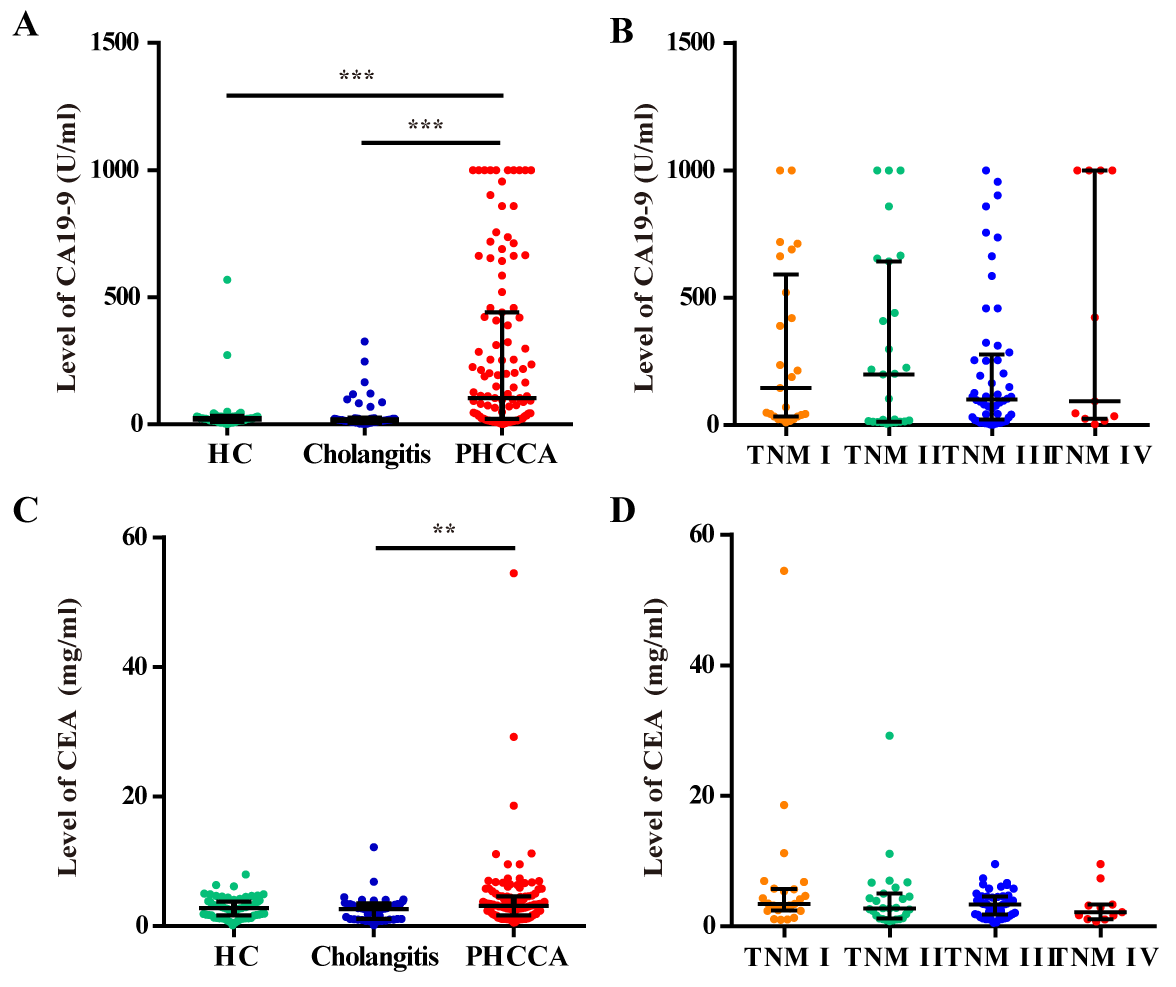

Supplement: Supplementary file 1 [file DataSheet_1.zip › supplementary materials/figure s4.tif]

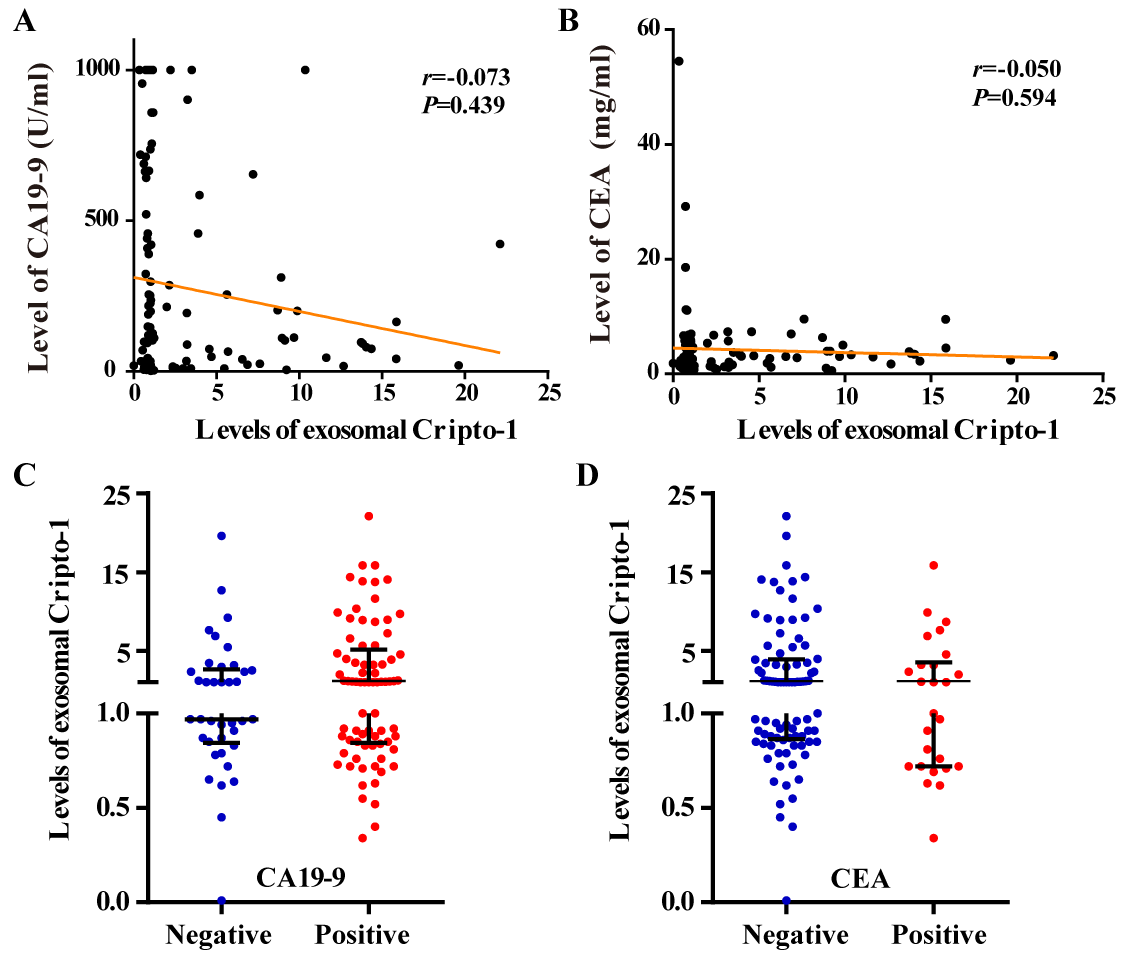

Supplement: Supplementary file 1 [file DataSheet_1.zip › supplementary materials/figure s5.tif]

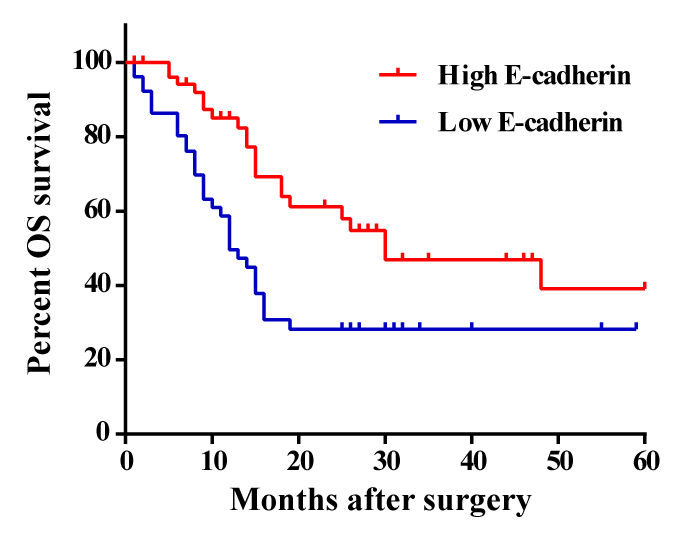

Supplement: Supplementary file 1 [file DataSheet_1.zip › supplementary materials/figure s6.tif]

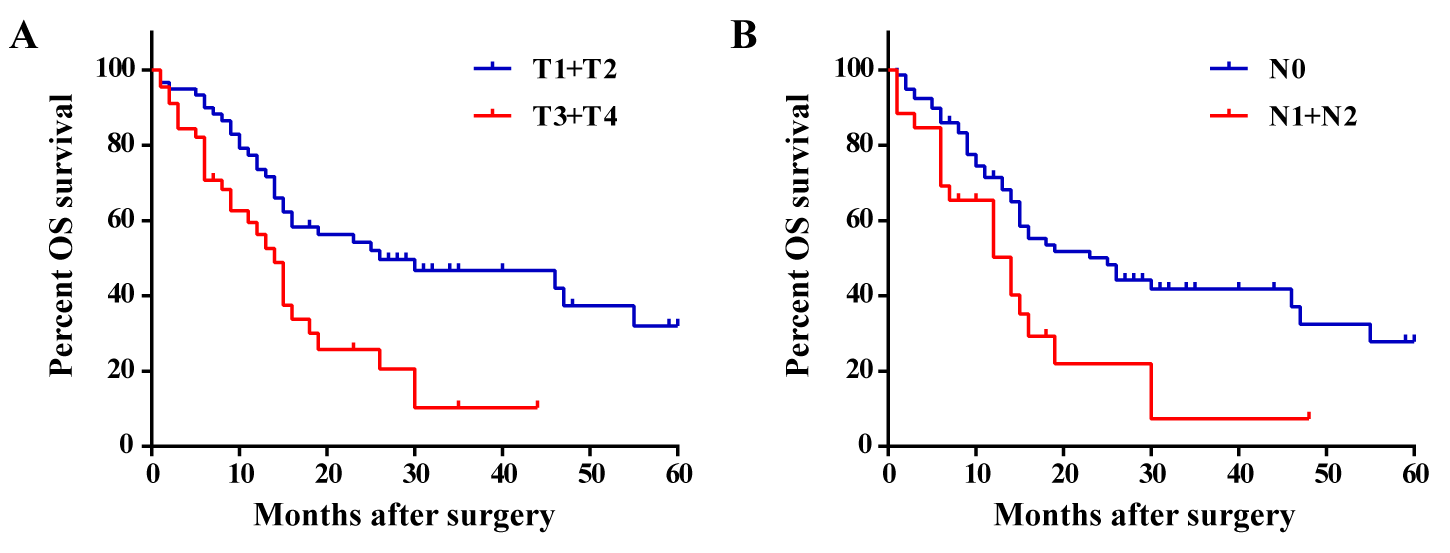

Supplement: Supplementary file 1 [file DataSheet_1.zip › supplementary materials/figure s7.tif]
