# Supplementary material for: Exosomal Cripto-1 Serves as a Potential Biomarker for Perihilar Cholangiocarcinoma
Source: Front Oncol. 2021 Aug 9;11:730615. doi: 10.3389/fonc.2021.730615 (PMC8380828; doi:10.3389/fonc.2021.730615)
Supplement: Supplementary file 1 [file DataSheet_1.zip › supplementary materials/Table s1.doc]

**Table S1.** Correlations between levels of exosomal Cripto-1 and clinicopathological characteristics

| Parameters | No. | Cripto-1 | *P*-value |
| --- | --- | --- | --- |
| Gender |  |  | 0.131 |
| Male | 75 | 1.01(0.83-3.21) |  |
| Female | 40 | 1.58(0.87-7.52) |  |
| Age |  |  | 0.068 |
| <65 year | 73 | 1.04(0.89-4.63) |  |
| ≥65 year | 42 | 0.96(0.78-3.05) |  |
| Differentiation |  |  | 0.433 |
| Well | 33 | 1.00(0.81-3.53) |  |
| Moderate | 64 | 1.06(0.88-3.96) |  |
| Poor | 18 | 0.96(0.81-4.87) |  |
| Tumor size |  |  | 0.120 |
| <3cm | 60 | 1.00(0.82-3.04) |  |
| ≥3cm | 55 | 1.05(0.87-6.87) |  |
| T stage |  |  | 0.124 |
| T1+T2 | 74 | 1.02(0.81-2.65) |  |
| T3+T4 | 41 | 1.15(0.86-7.19) |  |
| Lymph nodes metastasis |  |  | 0.007 |
| N0 | 84 | 0.97(0.82-2.87) |  |
| N1+N2 | 31 | 2.22(0.96-5.47) |  |
| Distant metastasis |  |  | 0.001 |
| No | 110 | 1.01(0.84-3.31) |  |
| Yes | 5 | 11.6(6.29-17.4) |  |
| Neural invasion |  |  | 0.074 |
| Negative | 84 | 1.01(0.80-3.25) |  |
| Positive | 31 | 1.24(0.88-8.69) |  |
