# Supplementary material for: Exosomal Cripto-1 Serves as a Potential Biomarker for Perihilar Cholangiocarcinoma
Source: Front Oncol. 2021 Aug 9;11:730615. doi: 10.3389/fonc.2021.730615 (PMC8380828; doi:10.3389/fonc.2021.730615)
Supplement: Supplementary file 1 [file DataSheet_1.zip › supplementary materials/Table s2.doc]

**Table S2.** Correlations between Cripto-1 expression and clinicopathological characteristics

| Parameters | No. of patients | Cripto-1  (high) | Cripto-1  (low) | *P*-value |
| --- | --- | --- | --- | --- |
| Gender |  |  |  | 0.281 |
| Male | 73 | 35 | 38 |  |
| Female | 32 | 19 | 13 |  |
| Age |  |  |  | 0.099 |
| <65 year | 51 | 22 | 29 |  |
| ≥65 year | 54 | 32 | 22 |  |
| Differentiation |  |  |  | 0.835 |
| Well | 23 | 13 | 10 |  |
| Moderate | 55 | 27 | 28 |  |
| Poor | 27 | 14 | 13 |  |
| Tumor size |  |  |  | 0.850 |
| <3cm | 59 | 28 | 31 |  |
| ≥3cm | 46 | 26 | 20 |  |
| T stage |  |  |  | 0.403 |
| T1 | 23 | 9 | 14 |  |
| T2 | 37 | 20 | 17 |  |
| T3 | 23 | 11 | 12 |  |
| T4 | 22 | 14 | 8 |  |
| Lymph nodes metastasis |  |  |  | 0.011 |
| N0 | 79 | 35 | 44 |  |
| N1+N2 | 26 | 19 | 7 |  |
| Neural invasion |  |  |  | 0.926 |
| Negative | 81 | 38 | 43 |  |
| Positive | 24 | 11 | 13 |  |
